# Classifying mental motor tasks from chronic ECoG-BCI recordings using phase-amplitude coupling features

**DOI:** 10.3389/fnhum.2025.1521491

**Published:** 2025-03-12

**Authors:** Morgane Marzulli, Alexandre Bleuzé, Joe Saad, Felix Martel, Philippe Ciuciu, Tetiana Aksenova, Lucas Struber

**Affiliations:** ^1^Clinatec, CEA, LETI, University Grenoble Alpes, Grenoble, France; ^2^CEA, LIST, University Grenoble Alpes, Grenoble, France; ^3^CEA, Joliot, NeuroSpin, Université Paris-Saclay, Gif-sur-Yvette, France; ^4^MIND Team, Inria, Université Paris-Saclay, Palaiseau, France

**Keywords:** brain-computer interface, electrocorticography, motor decoding, neural features, phase-amplitude coupling

## Abstract

**Introduction:**

Phase-amplitude coupling (PAC), the modulation of high-frequency neural oscillations by the phase of slower oscillations, is increasingly recognized as a marker of goal-directed motor behavior. Despite this interest, its specific role and potential value in decoding attempted motor movements remain unclear.

**Methods:**

This study investigates whether PAC-derived features can be leveraged to classify different motor behaviors from ECoG signals within Brain-Computer Interface (BCI) systems. ECoG data were collected using the WIMAGINE implant during BCI experiments with a tetraplegic patient performing mental motor tasks. The data underwent preprocessing to extract complex neural oscillation features (amplitude, phase) through spectral decomposition techniques. These features were then used to quantify PAC by calculating different coupling indices. PAC metrics served as input features in a machine learning pipeline to evaluate their effectiveness in predicting mental tasks (idle state, right-hand movement, left-hand movement) in both offline and pseudo-online modes.

**Results:**

The PAC features demonstrated high accuracy in distinguishing among motor tasks, with key classification features highlighting the coupling of theta/low-gamma and beta/high-gamma frequency bands.

**Discussion:**

These preliminary findings hold significant potential for advancing our understanding of motor behavior and for developing optimized BCI systems.

## 1 Introduction

Brain-computer interfaces (BCIs) devices are quickly transforming the field of motor rehabilitation. By establishing a direct communication pathway between the brain and external devices, BCIs enable individuals with motor impairments to control prosthetic limbs, digital assistants, and other assistive effectors using their brain activity. BCIs show potential to enhance the quality of life of people affected by strokes (Biasiucci et al., 2018), spinal cord injuries (Lorach et al., 2023), neurodegenerative diseases (Vansteensel et al., 2016), but can also open up new avenues for research on neuroscience and brain dynamics. Implanted BCIs based on electrocorticography (ECoG) or microelectrode arrays have achieved major advancements in recent years, both in their decoding capabilities (Benabid et al., 2019; Metzger et al., 2023; Deo et al., 2024) and their usability in home environments (Lorach et al., 2023; Vansteensel et al., 2024).

Decoding different motor states from neural signals remains one of the main challenges of BCIs to enable the control of external effectors. As of today, most of current motor BCI systems rely on signal amplitude modulations, in different frequency bands [generally mu/beta (Abiri et al., 2019) and gamma (Krusienski et al., 2011)], as decoding features. Research on the brain motor-related behavior has indeed been generally focusing on the study of amplitude variations (Cheyne et al., 2008; Cheyne and Ferrari, 2013; Jurkiewicz et al., 2006; Muthukumaraswamy, 2010; Saleh et al., 2010). In ECoG, multiple studies showed that high-gamma amplitude oscillations (>70*Hz*) correlate closely with specific aspects of motor functions (Tam et al., 2019; Wang et al., 2012; Branco et al., 2017; Spüler et al., 2014). While amplitude modulation based decoding is effective, it overlooks some dimensions of the neural signals. For example, the signal phase modulations, in particular in the very low frequency band (< 2*Hz*), also seem to contain information about motor behaviors (Combrisson et al., 2017). Currently only few—if any—BCIs use phase information as decoding features, which has however been shown to be informative for continuous movement decoding (Hammer et al., 2013).

To address these limitations, recent research has explored dynamic interactions between neural oscillations in different frequency bands, a phenomenon known as cross-frequency coupling (CFC). Among these, theta/gamma coupling is the most frequently reported and is thought to support different cognitive operations such as short-term memory, long-distance communication, and sensory stimulus integration (Lisman and Jensen, 2013; Hyafil et al., 2015). A particular type of CFC, phase-amplitude coupling (PAC) where the phase of a slow oscillation modulates the amplitude of a faster oscillation has also been studied in motor processes. PAC measurements have shown promise in highlighting critical neural patterns during movement execution and resting phase in ECoG data (Miller et al., 2012), and even during different phases of movement namely preparation and execution in EEG recordings (Combrisson et al., 2017). These studies suggest that PAC features could be used for motor states decoding in the context of BCI. Unlike most decoding methods that focus solely on signal amplitude across frequency bands, PAC-based decoding provides a more nuanced perspective by examining how the phase of low-frequency oscillations interacts with the amplitude of high-frequency oscillations. This interplay could provide insights on richer neural codes, which may contain information beneficial to BCI decoding (Canolty and Knight, 2010).

In this study, we investigate whether PAC-derived features (1) provide relevant information to differentiate various motor behaviors from the resting state in sensorimotor ECoG recordings and (2) could be used to in the context of asynchronous BCIs systems. We computed PAC from ECoG data acquired with WIMAGINE implant (Mestais et al., 2015) on a single tetraplegic individual performing attempted motor tasks to control a virtual avatar. To test their predictive power and gain understanding on how PAC is modulated across different attempted movements, these features were employed to train supervised classifiers to categorize hand movements from idle, as well as right from left hand movements. PAC features were then implemented in a pseudo-online manner for a 3-states classification, to assess if they could be used in real-time asynchronous ECoG-BCIs. The primary objectives of this study are to investigate the potential contribution of PAC features in decoding motor attempts from brain signals and better characterize PAC within the sensorimotor cortex during lateralized motor tasks. We show that PAC features achieve a high-accuracy classification between a motor attempt and rest, as well as between two distinct motor attempts. Additionally, we demonstrate that a PAC-based BCI can be implemented in a pseudo-online setup, although the decoding accuracy does not show significant improvement when compared to amplitude-based decoding.

## 2 Materials and methods

### 2.1 Data and participant

The dataset analyzed in this study was recorded as a part of the “BCI and Tetraplegia” clinical trial (ClinicalTrials.gov identifier: NCT02550522). The participant is a 28-years-old right-handed man with traumatic sensorimotor tetraplegia after a C4-C5 spinal cord injury (Benabid et al., 2019). He was implanted bilaterally above the left and right primary motor and sensory cortices with two WIMAGINE implants (Mestais et al., 2015), recording ECoG signal at a sampling rate of 586 Hz. Each implant consists of an 8 × 8 electrode grid, however due to the data transfer limit, only 32 electrodes organized on a checkerboard-like pattern were recorded on each implant, for a total of 64 electrodes. At the time of experiment, the subject was already experienced in BCI setup. Since his implantation, he gradually learned to use the BCI to control effectors with up to 8 degrees of freedom (Moly et al., 2022).

**Figure 1:**
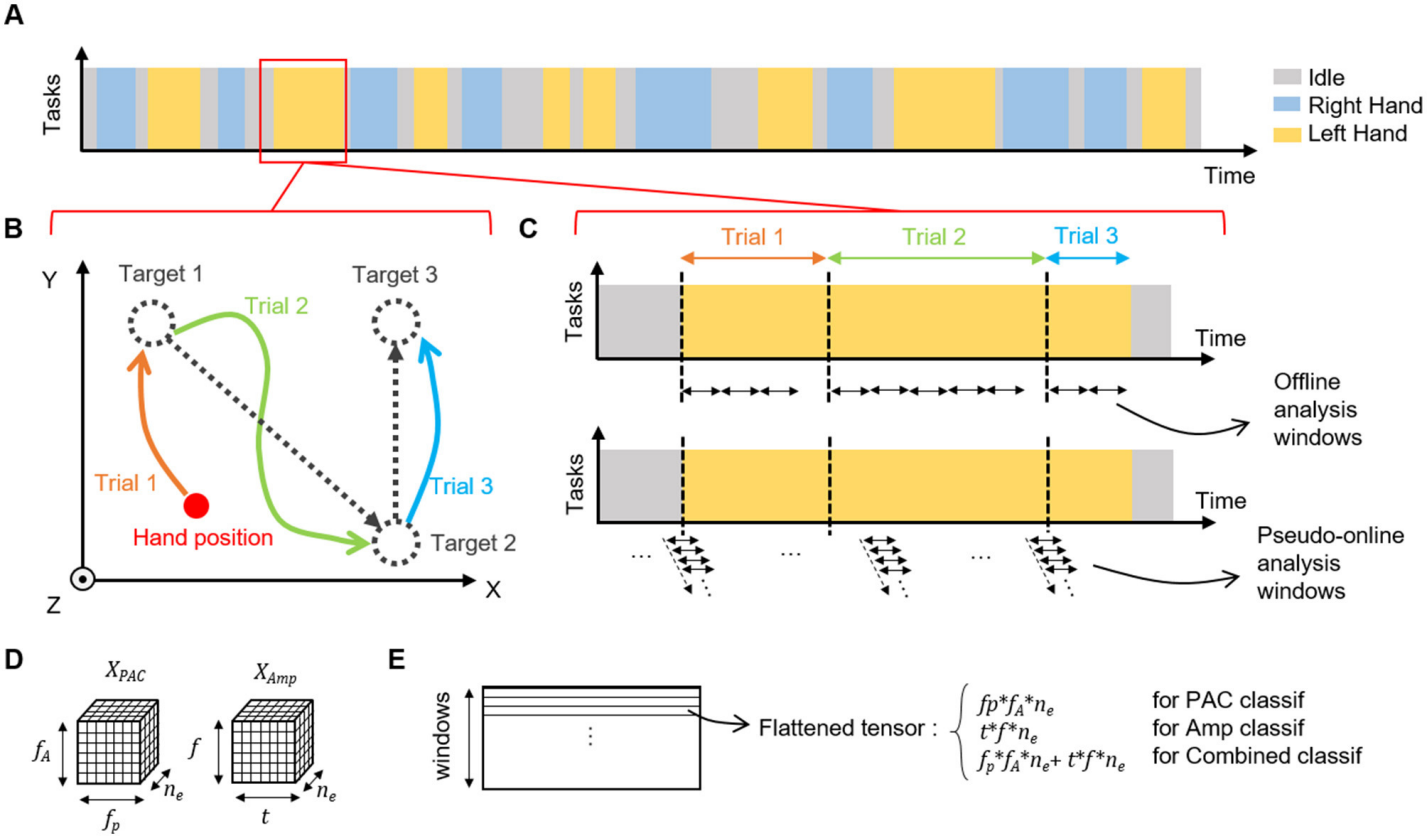
Experimental design. **(A)** Example of a session sequence of tasks. One session is composed of successive tasks of left hand, right hand or idle (wrist states that were not considered in this study are not represented). **(B)** Each active task (left or right hand) is composed of several trials in which the patient must reach the proposed targets in 3D. The cursor position is not reset between tasks, during task and during idle state. **(C)** Example of the timing of a task and organization of temporal windows for offline and online analysis. For offline analysis, non overlapping windows (1, 3, 5, 8, or 10 s) were used, and there was no window that overlapped between trials and/or tasks. In pseudo-online setup, 1 s windows with 90% of overlap were used allowing a 10 Hz prediction rate for the model. Windows were defined regardless of tasks or trials. **(D)** Shape of PAC and amplitude features tensors used in the offline and pseudo-online classifications. PAC tensor $X_{PAC}\in {\text{IR}}^{6\times 12\times 64}$, corresponding to the number of phase and amplitude bins in the PAC computation, and the number of electrodes. Ampltiude tensor $X_{Amp}\in {\text{IR}}^{10\times 15\times 64}$, corresponding to the number of time and frequency bins in the amplitude features extraction process, and the number of electrodes. One tensor is computed per time-window. **(E)** Before the classification process, tensors are flattened in row-major order into a one-dimensional vector. For classification based on combined features, flattened tensors of PAC and amplitude are concatenated.

In this study, 32 experimental sessions recorded over more than 200 days were considered. In the experiments, the tetraplegic patient performed attempted movements in order to move a 3D virtual avatar. In particular, the patient used a strategy in which he repeatedly attempted fingers, hands, and arms movements to control an avatar in a virtual environment. Each session was composed of a series of successive tasks decided by the experimenter. Each task corresponded to one of the four possible movements (left or right hand 3D translation or left or right wrist 1D rotation) or an idle state. During idle state, no target was presented to the subject who had to remain in a non-active state until the next instruction. During active tasks, the patient attempted to switch into the correct state in a self-paced manner and then reach the target locations that were presented one after another. A new target was displayed by the experimenter when the subject had reached the previous one leading to unequal movement time for each target (average movement time for left and right hand translation trials : 22.2 ± 4.1*s*). Organization of the experiment is depicted in Figure 1.

During online experiments, a Recursive Exponentially Weighted Markov-Switching Multi-Linear model (REW-MLSM) decoder was used. This decoder was previously described in Moly et al. (2022). In a nutshell, it consists of a hierarchical decoding, where several predictions from different regression models are computed in parallel to provide a trajectory of hand movement or a wrist rotation angle. Regression outputs are then weighted by the output of a classifier based on a Hidden Markov Model (HMM) approach. The decoding model was trained online in closed-loop on the first four sessions, then the weights of the model were fixed and used to evaluate the performances on the remaining sessions. For the purpose of this study, calibration and test sessions were concatenated and only classification between 3 states was considered, idle (ID), left and right hand translations (respectively LH and RH). The final dataset was globally well balanced between tasks and consisted of around 18 h of experiment, comprising 11.3 min, 11.0 min, and 11.2 min per session on average for ID, LH and RH states, respectively.

### 2.2 Phase-amplitude coupling features extraction

The ECoG signal was mapped in the time-frequency domain by convolution with Morlet waveforms (Complex Continuous Wavelet Transform - CCWT). Central frequencies of the waveforms ranged from 5 to 30 Hz with a 2.5 Hz step (11 steps), as a range of interest for phase modulating frequencies (*f*_*p*_), and from 30 to 150 Hz with a 10 Hz step (13 steps), as a range of interest for amplitude modulated frequencies (*f*_*A*_), for all the electrodes (64 in total, 32 from left implant and 32 from right implant). The range of frequencies considered has been chosen to represent the theta, alpha, beta bands for the phase, and gamma for the amplitude to study all the possible couplings. The modulus (respectively, the argument) of the CCWT outputs represents the instantaneous amplitude (respectively, phase) information required to compute the PAC.

Several methods have been proposed in the literature to quantify PAC. These methods differ in their theoretical foundations, and it was shown that the modulation index (MI) (Tort et al., 2008) and the mean vector length (MVL) (Canolty et al., 2006; Okurt and Schnitzler, 2011) are the most suitable in terms of accuracy while keeping a good computational efficiency (Hlsemann et al., 2019; Tort et al., 2010). Here, we used both MI and MVL measurements to combine their respective strengths and weaknesses and compare their qualitative estimation of PAC in ECoG recordings.

MI quantifies the deviation of a phase-amplitude distribution from the uniform one using an adapted Kullback-Leibler (KL) distance (Tort et al., 2008, 2010). The phase-amplitude distribution P is calculated by binning phases from the modulating frequency range *f*_*p*_ into a chosen number of bins (18 in Tort et al. (2008), kept in our study), and computing the normalized mean amplitude from the modulated frequency range *f*_*A*_ for each bin:


$$\begin{array}{l} P\left(j\right)=\frac{<A_{f_{A}}{>}_{\phi_{f_{p}}}\left(j\right)}{\overset{N}{\underset{k=1}{\sum }}<A_{f_{A}}{>}_{\phi_{f_{p}}}\left(k\right)}, \end{array}$$

where *A*_*f*_*A*__(*t*) and ϕ_*f*_*p*__(*t*) are the time series of the amplitudes and phases of the frequency ranges of interest, N the number of bins and < > denotes the mean over time. Then MI, ranging from 0 (no coupling) to 1 (strong coupling), is calculated as follows:


$$\begin{array}{l} MI=\frac{D_{KL}\left(P\right)}{log\left(N\right)}, \end{array}$$

where:


$$\begin{array}{l} D_{KL}\left(P\right)=log\left(N\right)-H\left(P\right),\\ H\left(P\right)=-\overset{N}{\underset{j=1}{\sum }}P\left(j\right)log\left[P\left(j\right)\right] \end{array}$$

with *H*(*p*) being the Shannon entropy of the distribution *P*.

MVL is a more straightforward way of estimating PAC (Canolty et al., 2006) as it is not based on distributions, but is directly calculated from amplitude and phase time series *A*_*f*_*A*__(*t*) and ϕ_*f*_*p*__(*t*). Given the time series of amplitudes and phases, MVL estimates the length of the mean vector of all the vectors in the polar plane represented by each complex number. If the magnitude of some of the vectors (amplitudes) is increased at a particular angle (phase), then the MVL has a non-zero value that represents the amount of PAC. To tackle one of the main drawbacks of MVL, which is its amplitude dependency, we computed the direct MVL proposed later by Okurt and Schnitzler (2011) with the following equation :


$$\begin{array}{l} MVL=\left|\frac{\overset{n}{\underset{t=1}{\sum }}A_{f_{A}}\left(t\right)e^{j\phi_{f_{p}}\left(t\right)}}{n}\right|. \end{array}$$

It normalizes the MVL using the amplitude, which bounds its values between 0 and 1 and renders it less vulnerable to high frequency band power variations.

Given that the measurement of the PAC is highly dependent on the duration of the window over which it is calculated, particularly for short time windows (Dvorak and Fenton, 2014), the amplitude and phase time series of each trial (one target reaching) were segmented into non-overlapping time-windows of equal size before estimating the PAC (either MVL or MI). Part of trials that were shorter than window length were excluded from this analysis (Figure 1). Furthermore, to assess the influence of window length on classification accuracy, windows of 1, 3, 5, 8 and 10 sec were considered. For each window, a phase-amplitude comodulogram of MI or MVL values was obtained using the amplitudes from the *f*_*A*_ bands *A*_*f*_*A*__(*t*) and the phases from the *f*_*P*_ bands ϕ_*f*_*p*__(*t*), for the whole window duration. PAC was computed for each frequency band pair in (*f*_*A*_, *f*_*p*_), using all the combinations of *A*_*f*_*A*__(*t*) and ϕ_*f*_*p*__(*t*), as no preliminary assumption was drawn on the modulating and modulated frequencies. The same compute process was repeated for each electrode, leading to a PAC features tensor $X_{PAC}\in {\text{IR}}^{11\times 13\times 64}$ (11 bands for phase, 13 bands for amplitude, 64 channels) for each window (Figure 1).

### 2.3 Offline binary classification

To assess whether PAC measurements contained information related to the patients individual mental tasks, we conducted three different binary classification tests based on PAC features: idle vs right hand (ID-RH), idle vs left hand (ID-LH) and right hand vs left hand (RH-LH).

The classification was performed with flattened (reshaped in row-major order into a one-dimensional vector) PAC features using two different classifiers: a Partial Least Square regression (PLS) with output variable encoding for classification and a Linear Discriminant Analysis (LDA). PLS was chosen because it is particularly suited for cases with high dimensional features space. In addition it is similar to the BCI classification we use in online settings. LDA was used as a more classical approach for classification, to validate the conclusion drawn from PLS.

PLS extracts a set of latent variables or components that capture the maximum covariance between the independent and dependent variables, and then, constructs a regression model by sequentially fitting these components to the data (Geladi and Kowalski, 1986). To use PLS for classification, the response variables were encoded as *n*×*m* binary matrix (*m* = 2 being the number of classes), with the desired class (i.e. ID, RH and LH) labeled as 1 and the other as 0. The regression output was then discretized by selecting the class with the maximum predicted value as the predicted class. This approach facilitated the modeling of the relationship between predictor variables and class labels. To find the optimal number of components to use in this PLS, other PLS classifications were conducted on a smaller dataset that was recorded before this experiment in which the subject was performing a similar but slightly different task. For this dataset, classification was repeated for each number of components from 1 to 15, and the performance metrics estimated with a 10-fold cross-validation scheme (i.e., mean squared error and percentage of explained variance) were averaged across the folds. The number of components was selected as the best tradeoff between minimal error and maximum explained variance, which led to 6 components in the initial PLS (see Supplementary Figure S1).

Linear Discriminant Analysis (LDA) is a classification and dimensionality reduction technique that projects data onto a space that maximizes class separability. It finds a linear combination of features that best distinguishes between classes by maximizing between-class variance and minimizing within-class variance (Tharwat et al., 2017).

The classification output performance of both classifiers was evaluated on a 5-fold stratified cross validation scheme with balanced accuracy to take into account potential class imbalances within sessions. Although the database is globally balanced, some sessions have individually a slight imbalance. Balanced accuracy was computed for each fold and average across folds, as follows:


$$\begin{array}{l} \text{balanced accuracy}=\frac{1}{2}\frac{TP}{TP+FN}+\frac{1}{2}\frac{TN}{TN+FP}, \end{array}$$

with TP, FN, TN and TP respectively the number of true positives, false negatives, true negatives and false positives.

For each pair of tasks and classifier, the obtained balanced accuracies across sessions were statistically compared using two-way ANOVAs, with the first factor being the used PAC value (MI or MVL) and the second factor the length of the time-window (from 1 to 10 seconds). Since the distributions of the residuals were not normal, ANOVAs were performed after a rank-transformation procedure on balanced accuracies.

### 2.4 Features analysis

To evaluate the importance of the features in the classification process of the PLS, in which components are obtained as linear combinations of the original variables, the VIP (Variable Influence on the Projection) score of each feature was computed. VIP score measures the importance of each variable in explaining the variance of the response variable (Y) in the PLS model. The VIP computation is based on the PLS weights, weighted by how much of the responses are explained by each PLS component:


$$\begin{array}{l} VIP_{k}=\sqrt{\frac{K\cdot \overset{A}{\underset{a=1}{\sum }}w_{ak}^{2}SSY_{a}}{A\cdot SSY_{total}}}. \end{array}$$

where *K* is the number of original predictors, *A* the number of PLS components, *w*_*ak*_ is the weight of the *k*^*th*^ feature in the *a*^*th*^ component, *SSY*_*a*_ the amount of sum of squares of Y explained by the *a*_*th*_ component and *SSY*_*total*_ the amount of sum of squares of Y explained by all components (Wold et al., 1993). Variables with VIP scores greater than 1 are typically considered important.

The VIP score was used to study the features from a spectral and spatial point of view. First, the importance of different frequencies and frequency-couples in the classification was assessed by the number of occurrences of a VIP score greater than 1. Then, for the most represented frequency pair, we depicted the mean VIP score over each electrode of the implant to analyze the localization of the features of importance.

Features of importance analysis was only performed for PLS-based classification as it output better classification results than LDA, then should provide more accurate insights on spectral and spatial contributions.

### 2.5 Shared variance between gamma amplitude and PAC

To assess on which extent information provided by PAC was independent from the information contained in amplitude of gamma time series, we estimated the portion of variance of gamma amplitude that can be explained by MVL. To do so, for each window on which MVL was calculated, values of gamma amplitude were extracted by averaging *A*_*f*_*A*__(*t*) time series over window duration. This led to a tensor of gamma amplitude $X_{\gamma }\in {\text{IR}}^{13\times 64}$ (13 bands, 64 electrodes). Then, for each session, each band and each electrode, a linear model was fitted between gamma amplitude and PAC estimated by MVL method, which was averaged over all phase frequency bands. For each model, the coefficient of determination *R*^2^ was assessed as the proportion of the total sum of squares explained by the model.

### 2.6 Pseudo-online classification

Previous offline method using non overlapping and long windows is inapplicable in an online asynchronous BCI. Then, to assess if PAC features could be used in online asynchronous BCI settings, classification was also performed in a pseudo-online manner. PAC features were computed in the same way as the previous binary-classification approach, obtaining the time series *A*_*f*_*A*__ and ϕ_*f*_*P*__ in the frequency bands of interest. However, instead of considering windows containing only data from the same trial and task, PAC features $X_{PAC}\in {\text{IR}}^{11\times 13\times 64}$ (11 bands for phase, 13 bands for frequency, 64 channels) were computed every 0.1 s, based on the last second of signal (sliding window with 0.9 s overlap), simulating an online feature computation at the rate of 10 Hz.

Similar to online experiments, Hidden Markov Models (HMM) combining emission and transition probabilities were trained and used for 3-class classification (ID, RH, LH) in a pseudo-online setup (Moly et al., 2022). Emission probability was computed using Recursive Exponentially Weighted N-way Partial Least Squares Regression (REW-NPLS) with one-hot encoded class labels, post-processed by softmax function. REW-NPLS is a variant of PLS for tensor features and compatible with recursive online updates (Eliseyev et al., 2017). The transition probability matrix was estimated by counting the number of transitions in the training set. The class prior was established to ensure equal probability distribution among classes.

To assess if PAC features add information to the amplitude features generally used, the 3-class pseudo-online classification task was performed based on PAC features only, on amplitude features only and on the combination of PAC and amplitude features concatenated in the same tensor. For amplitude features the same CCWT as for PAC features computation was used to map the ECoG signal in the time-frequency domain, with central frequencies ranging from 10 to 150 Hz (10 Hz step, 15 bands). Features were then defined as 10 points description of 1-s windows of the amplitude time series (averaging over 0.1 s fragments) with 0.9 s overlap, leading to an amplitude features tensor $X_{Amp}\in {\text{IR}}^{10\times 15\times 64}$ (10 time points, 15 bands, 64 channels) for each window. Note that these are the same features that were used during the online experiments (Moly et al., 2022). To combine PAC and amplitude features in the same classification, tensors were flattened and concatenated over time and frequency dimensions resulting in a combined features tensor $X_{Both}\in {\text{IR}}^{222\times 64}$.

As the MVL method showed better performances than the MI in offline binary classifications, only the MVL was used to compute PAC features for the pseudo-online setup. Models were retrained on the first six sessions, which allows to reach 85% of the maximum achievable performances, defined as a REW-MLSM decoder trained on all sessions (Śliwowski et al., 2023).

The obtained models were then tested on every other sessions and balanced accuracy was calculated. For each class, accuracy (one-vs.-all), recall, precision and specificity were also assessed as follows:


$$\begin{array}{l} \text{accuracy}=\frac{TP+TN}{TP+TN+FP+FN},\\ \text{recall}=\frac{TP}{TP+FN},\\ \text{precision}=\frac{TP}{TP+FP},\\ \text{specificity}=\frac{TN}{TN+FP}. \end{array}$$

Chance levels were estimated by computing the same metrics 100 times with random permutations of the real label vector, then averaging the results across repetitions.

Weights of the amplitude and PAC models were were also analyzed to determine whether classifications were based on the same spatial patterns and frequency bands. To do so, model regression tensors were summed over all dimensions except the dimension of interest.

All the analysis were performed with Matlab R2024a. In the offline classifications, the MI and MVL indices were computed with the original codes proposed by the respective authors (Tort et al., 2010; Okurt and Schnitzler, 2011). In the pseudo-online classification, the MVL computation was performed in a tensor-based way adapted from Tensorpac (Combrisson et al., 2020).

## 3 Results

### 3.1 Offline binary classifications: performances

**Figure 2:**
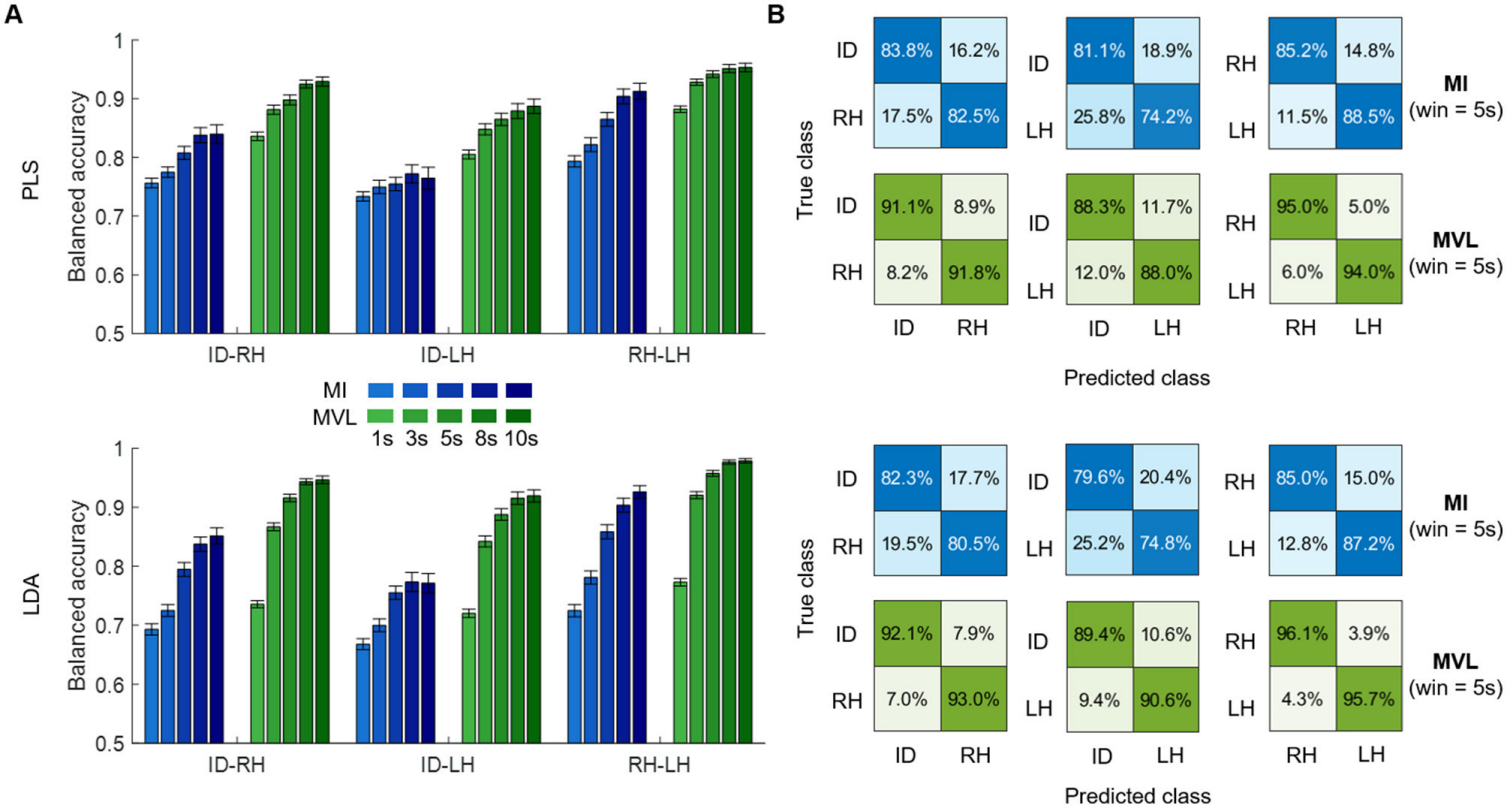
Binary classification results. **(A)** Average balanced accuracy of PLS (top) and LDA (bottom) using MI (blue) or MVL (green) features computed on different time windows lengths (shaded) for three different tasks: idle vs. right hand (ID-RH), idle vs. left hand (ID-LH) and right vs. left hand (RH-LH). **(B)** Confusion matrices (row normalized) depicting results of the 5s window length case.

We tested MI and MVL features in three classification tasks (ID-RH, ID-LH, RH-LH) with two different classifiers and several time windows. Although both features sets performed above chance (33% in balanced accuracy), MVL demonstrated significantly higher performances regardless of the classification task, the classifier used and the length of the time window (*p* < 0.001—Figure 2A). Regarding the influence of the time window, a significant loss of performance was observed as the window length decreases, more prominent when using LDA as a classifier as opposed to PLS (*p* < 0.001 both for PLS and LDA). The higher accuracy was reached for 10 s-windows with 92.9 ± 0.7% (mean ± sem), 88.7 ± 1.2% and 95.3 ± 0.7% respectively for ID-RH, and RH-LH classifications using PLS and 94.6 ± 0.6%, 91.9 ± 1.0% and 97.9 ± 0.4% respectively for ID-RH, for ID-RH, ID-LH and RH-LH classifications using LDA. This accuracy dropped to 83.6 ± 0.7%, 80.5 ± 0.7% and 88.2 ± 0.6% respectively for ID-RH, ID-LH and RH-LH classifications using PLS and 73.6 ± 0.6%, 72.0 ± 0.7% and 77.3 ± 0.6% respectively for ID-RH, ID-LH and RH-LH classifications using LDA, when window length was reduced to 1 s.

These results illustrate that MVL features are more efficient for classifying ECoG motor tasks on a span of a few seconds, as also illustrated by the average row-normalized confusion matrices for a 5s window (Figure 2B).

### 3.2 Offline binary classifications : features' importance

To assess the impact of different features components on classification performance, we conducted VIP score analysis, both on spectral and spatial perspectives. Since results were similar across window lengths, only those with a 5 s window are presented here.

**Figure 3:**
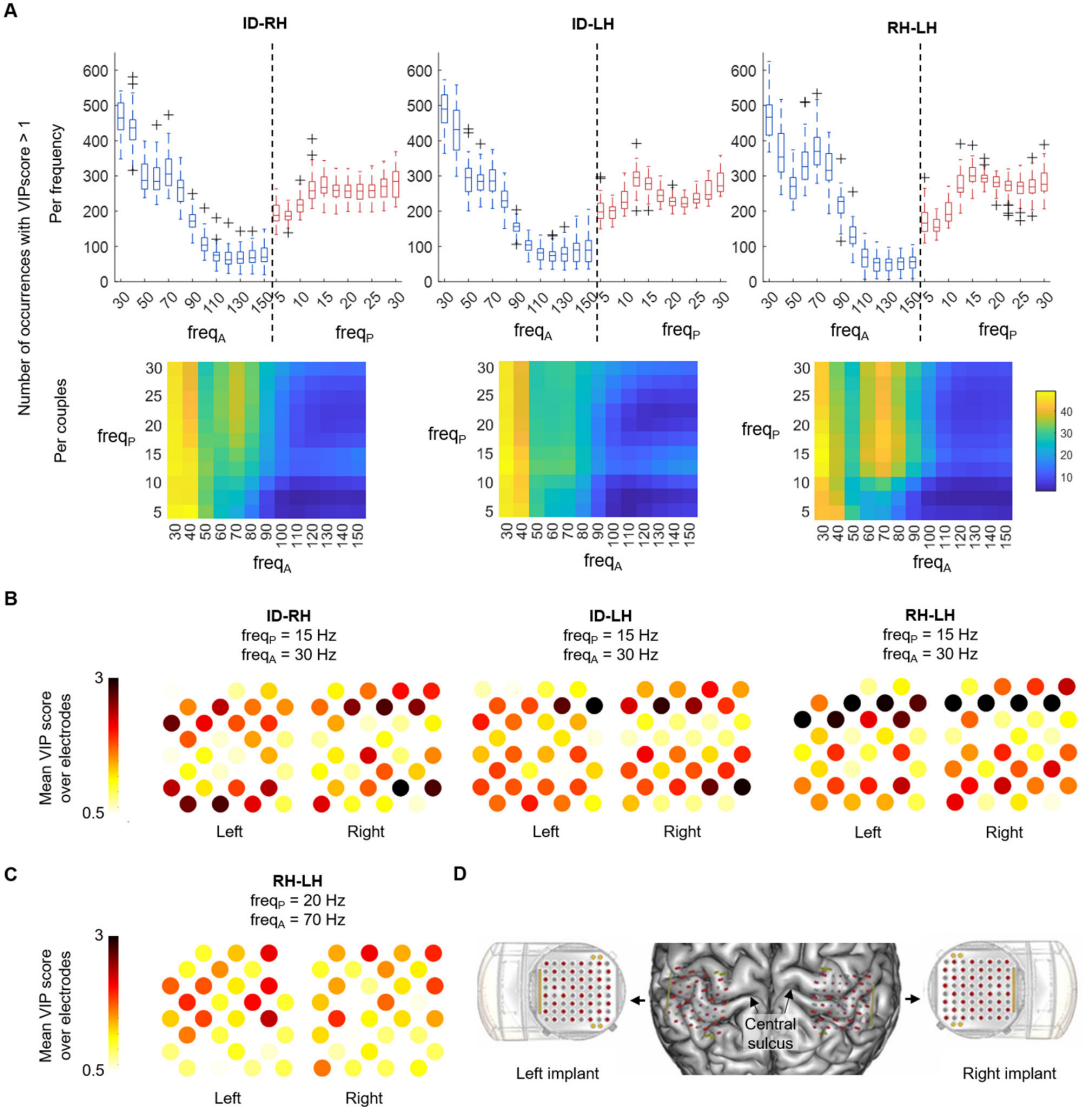
PAC spectral and spatial features analysis, with a 5 s window. **(A)** Top: boxplot of the number of occurrences of the different frequencies with a VIP score > 1. On each box, the central mark indicates the median of occurrences of amplitude (blue) and phase (red) frequencies of interest in the features; the bottom and top edges of the box indicate the 25th and 75th percentiles, respectively. Bottom: occurrences are shown by amplitude-phase frequencies couples. **(B)** Average VIP scores of each WIMAGINE implant electrode following their anatomical distribution for the three classification scenarios for the 15-30 Hz phase-amplitude couple. **(C)** Average VIP scores of each WIMAGINE implant electrode following their anatomical distribution for the RH-LH case, for the 20–70 Hz couple. **(D)** Anatomical localization of the implants and the electrodes on the brain surface reconstruction of the subject.

For all the classifications, the gamma band (30–80 Hz) was the most modulated amplitude while the phase frequencies were all represented in the PLS weights. However, looking at the most represented couples highlighted different pattern of contributing frequencies in the distinction of *idle vs. hand* and *hand vs. hand* tasks (Figure 3A). In the ID-RH and ID-LH classifications, the most contributing phase-amplitude couplings were the modulation of the amplitude of the 30–40 Hz low-gamma band by the whole range of frequency bands for phase (5–30 Hz) (e.g., 43.0 ± 0.8 and 45.7 ± 0.9 occurrences of the 15–30 Hz PAC for ID-RH and ID-LH respectively). This coupling, which was maximal at 15 Hz and below, was also present in RH-LH classification (45.0 ± 0.9 occurrences of the 15–30 Hz PAC), however a pattern of beta/high-gamma coupling also figured in the comodulogram with the modulation of the 70 Hz amplitude by the 15 -30 Hz phase (e.g. 40.8 ± 1.1 occurrences of the 20 Hz–70 Hz PAC).

Figures 3B, C show the anatomical spatial distribution on the left and right implants of electrodes of importance, based on their average VIP scores. The VIP scores are shown for the influential amplitude-phase frequency couples found in the previous analysis for the different classification cases (15–30 Hz for ID-RH, ID-LH and RH-LH in Figure 3B and 20–70 Hz for RH-LH in Figure 3C). For the 15–30 Hz coupling, the pattern of contributing electrodes was similar across the classification tasks with contribution of large clusters of electrodes, in particular on the anterior and posterior parts of the implants (mean VIP score, all electrodes, ID-RH: 1.38 ± 0.51 and 1.42 ± 0.54; ID-LH: 1.41 ± 0.45 and 1.55 ± 0.48; RH-LH: 1.48 ± 0.62 and 1.58 ± 0.72 for left and right implants respectively). Regarding the 20–70 Hz coupling present int the RH-LH case, it can be observed that contributing electrodes are less clustered, with information extracted from few individual electrodes (mean VIP score, all electrodes, left implant: 1.20 ± 0.40, right implant: 1.19 ± 0.32). Figure 3D provides the anatomical localization of the implants on the subject's cortex.

### 3.3 Shared variance between PAC and gamma amplitude

**Figure 4:**
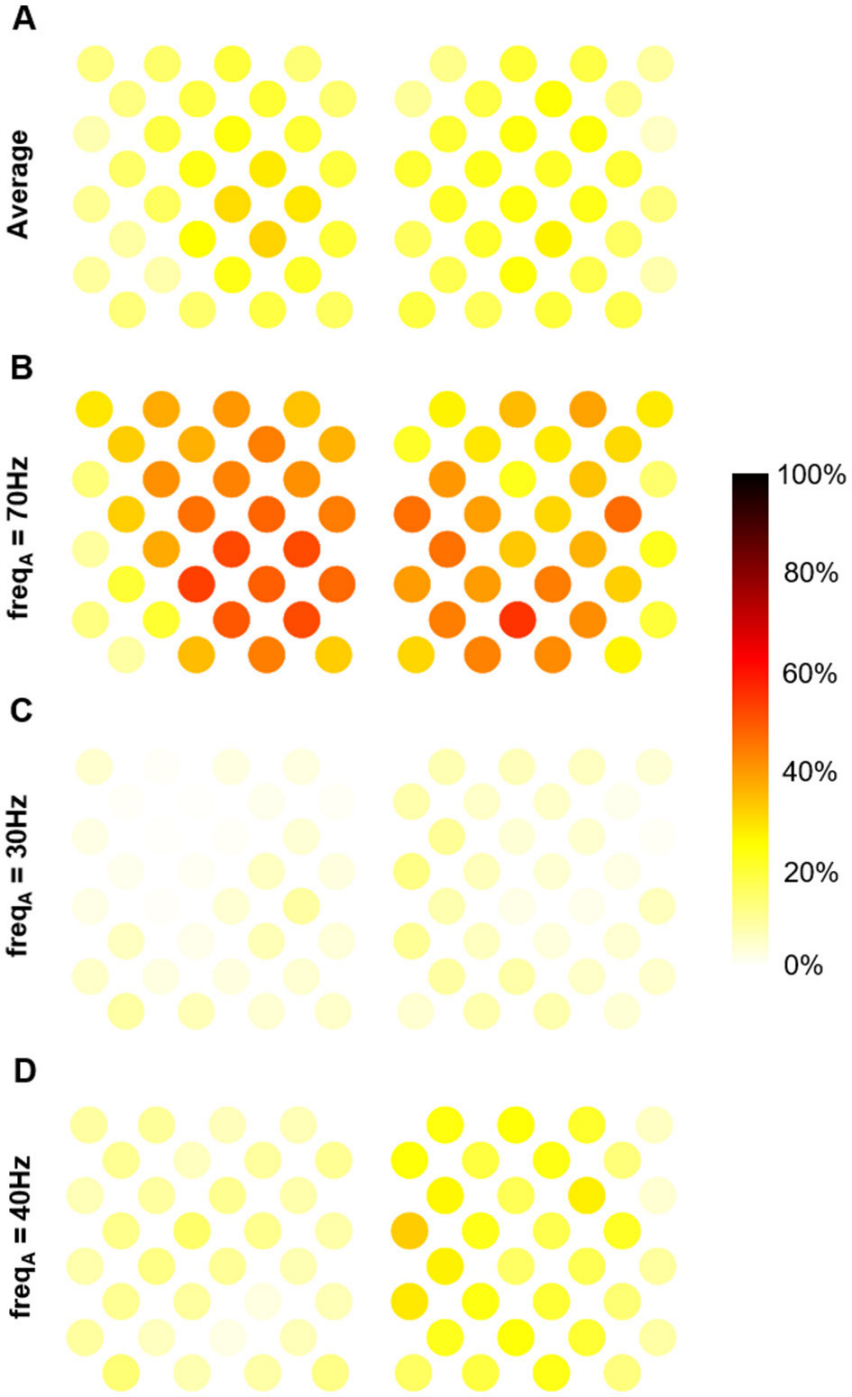
Portion of shared variance between MVL-based PAC and gamma amplitude averaged over time window (with a 5s time-window). **(A)** Average over frequencies. **(B)** At 70 Hz (maximal shared variance). **(C)** At 30 Hz (minimal shared variance). **(D)** At 40 hz.

Since PAC can be used to decode motor states, like gamma amplitude (Branco et al., 2017), we may ask whether the information carried by these two markers is identical, distinct, or overlapping. To assess the extent to which these two pieces of information overlap, we conducted a regression analysis. Figure 4 shows the coefficient of determination (portion of shared variance) between PAC and gamma amplitude across the electrodes for different frequency bands, using a time window of 5 s. On average across frequencies, PAC accounted for 6 to 31% of gamma amplitude variance for the different electrodes (Figure 4A). The portion of shared variance was maximal at 70 Hz, ranging from 9 to 50% across the implant (Figure 4B), and minimal at 30 Hz ranging from 0 to 12% (Figure 4C).

### 3.4 Pseudo-online classification results

**Figure 5:**
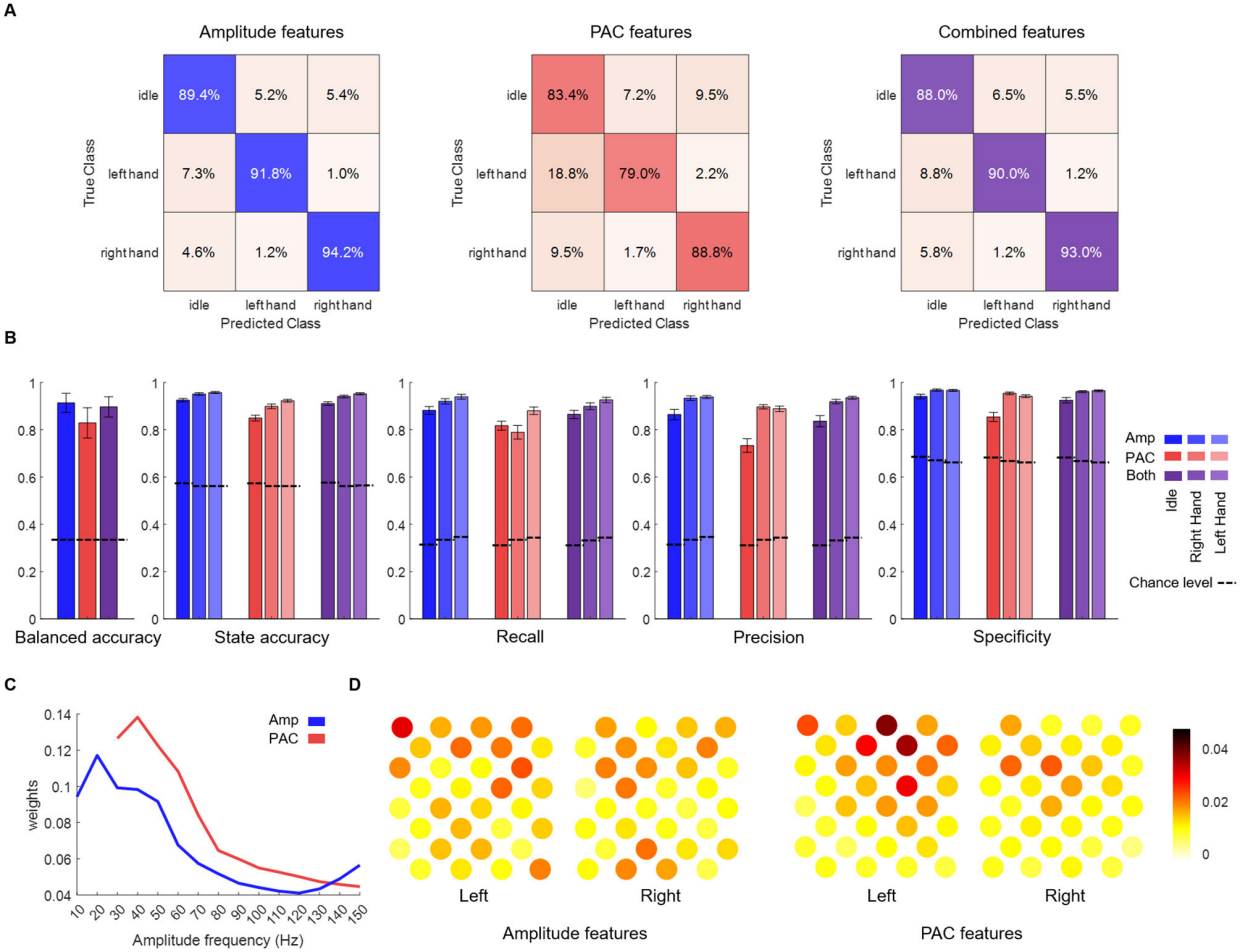
Pseudo online classification results.**(A)** Confusion matrices (row normalized) using the different input features. **(B)** Average balanced accuracy, accuracy (one vs. rest), recall, precision and specificity. Except for balanced accuracy, metrics were calculated per state. Performances of the pseudo-online classification are shown for amplitude (blue), PAC (red) or both (purple) features on the three states classification. States are shown in different shades. **(C)** Amplitude (blue) and PAC (red) models weights across amplitude frequency bands (weight tensors summed over time steps and electrodes for amplitude and over phase frequency bands and electrodes for PAC). **(D)** Amplitude and PAC models weights across electrodes (weight tensors summed over time steps and frequency bands for amplitude and over phase and amplitude frequency bands for PAC).

Lastly, we tested the PAC features performances in a pseudo-online setting (Figure 5), to compare their classifying power with the amplitude-only features. Classifications were performed using PAC features, amplitude features and the combination of PAC and amplitude features to test if information provided by PAC is different from that provided by amplitude. While PAC features showed a good classification power, amplitude features performed better (balanced accuracy by session) and combined features classification was not significantly different than amplitude-based (balanced accuracy by session 91.4% ± 4.0%, 82.9% ± 6.4% and 89.7% ± 4.3% for amplitude, PAC and combined features respectively). Interestingly, the confusion matrices (Figure 5A) reveal that misclassifications primarily occur when motor states (LH and RH) are incorrectly identified as idle. Thus, regarding precision and specificity, PAC features performed almost as well as amplitude-based features for right and left hands states, but with a lower recall. Regarding idle state precision/specificity and recall were lower with PAC features. Although combined features decoding did not outperform amplitude-based decoding, weights of the amplitude and PAC models indicates that decoding was based on overlapped but different frequency bands. The most contributing frequency band was centered on 20 Hz for amplitude decoding and on 40 Hz for PAC. Regarding spatial patterns, PAC decoding was based on more localized areas than amplitude decoding.

## 4 Discussion

The findings presented above advance empirical evidence of the discriminative power of phase-amplitude coupling measurements in neural oscillations to classify motor attempts of different movements. On one hand, we demonstrated that PAC enables offline classification of a motor attempt vs. rest, or between two different motor attempts, with high accuracy. However, this accuracy deteriorates as the length of the time window used for PAC computation decreases. On the other hand, we demonstrated that a PAC-based pseudo-online BCI operates effectively, although it is less efficient than amplitude-based decoding.

### 4.1 PAC features are able to decode motor attempts with high accuracies

The high classification accuracies achieved using PAC features derived from Mean Vector Length (MVL) in the offline approach highlights its efficacy in distinguishing motor attempts. This is in line with previous studies (Combrisson et al., 2017; Yanagisawa et al., 2012) that highlighted the capability of PAC features to discriminate different motor states and intentions, and demonstrates that PAC can effectively differentiate attempted motor tasks in ECoG. Although Modulation Index (MI), which also quantifies phase-amplitude coupling allowed to classify left and right hands and hand vs. idle, it showed significantly lower performances. This contrasts with previous research that showed the robustness of MI (Canolty et al., 2006; Tort et al., 2010) and suggest that MI is less suited for ECoG-BCI applications. This might be due to the fact that MVL is more sensitive to modulation strength high signal-to-noise ratio conditions (Hlsemann et al., 2019), which is the case with ECoG recordings as opposed to EEG.

The supervised machine learning framework based on PLS method also allowed to determine which PAC features (phase-amplitude couples and electrodes) contributed the most to the discrimination between the different states and motor tasks, as evaluated by features VIP scores. Furthermore, regression analysis showed that, although a portion of variance was shared between PAC and amplitude of gamma oscillations, they also carried out different information. In particular, while coupling with low-gamma amplitude was the major contributor discriminating *motor* and *idle* states, there was almost no shared variance between PAC and gamma amplitude in this frequency band. This suggests that classification framework was able to extract PAC information that was not present in gamma amplitude alone. In the contrary, at 70 Hz an important part of the variance was shared between PAC and gamma amplitude, although the distribution over the implant was different. Thus, classifier seems to have extracted information that is specific to PAC measure. Moreover, one should note that a part of the shared variance can be explained by the fact both measures correlated to the task (motor attempts). This does not necessarily mean that both measures encompass the same information.

### 4.2 Coupling with low-gamma amplitude discriminates motor and idle states

In all the binary classification evaluated, whether between *idle* and *motor* states (ID-RH, ID-LH), or *motor* and *motor* states (RH-LH), the coupling of the whole range of frequencies with low-gamma band contributed to the discrimination between states. It includes theta/low-gamma, alpha/low-gamma and to a lesser extent beta/low-gamma couplings. The spatial contributions were similar across classification tasks, and did not reveal a clear contralateral pattern when distinguishing *idle* from *motor* states, as it might have been expected. Instead, the coupling was visible widely over both implants. However, this coupling was also present in discriminating left from right *motor* states, indicating that the spatial pattern is nevertheless task-dependent.

Theta/gamma coupling, where the phase of theta oscillations drives the amplitude of the gamma oscillations is thought to be a neural code that reflects the coordination of the communication between brain regions (Lisman and Jensen, 2013). It has recently gained interest in cognitive neuroscience and has been observed across a wide variety of paradigms, in particular in tasks involving memory processes (Mormann et al., 2005; Sauseng et al., 2009; Axmacher et al., 2010) but also in sensorimotor tasks (Canolty et al., 2006). Thus, although brain activity of the patient was only recorded over the sensorimotor cortex, it is likely that the theta/gamma coupling we observed indicates large-scale integration and/or transmission of information from/to other brain areas involved in motor process. This can explain the wide spatial distribution of the contributing electrodes in the classifications, as well as the implication of both motor and sensorimotor cortices in the process. Low-gamma power has also been shown to be phase-locked to alpha oscillations, during resting spontaneous brain activity (Osipova et al., 2008). This phenomenon could also have contributed to the distinction between states.

During movement and motor attempts, theta and alpha activity, typically ranging from 4-8 Hz and 8-12 Hz, is particularly notable in the frontal and motor areas of the brain, where it is thought to play a role in motor planning and execution, enhancing the integration of sensory and motor information necessary for movement (Horschig et al., 2015; Brauns et al., 2014). In particular, studies have shown that theta rhythms are involved in the coordination of motor tasks, with increased event-locked theta activity observed in the following milliseconds (aroud 500 ms) after cues are presented to the subject - either motor preparation or motor execution cues (Pellegrino et al., 2018; Struber et al., 2021). This burst of activity then decreases during motor execution, as well as the theta/gamma coupling which has been shown to be accurately time-locked to the theta band activity evolution (Canolty et al., 2006). Alpha activity which is suppressed during movement, is also phase-locked to motor or execution cues and coupled with gamma band power albeit in a lesser extent (Canolty et al., 2006; Struber et al., 2021).

It is probable that our classifiers detected these brief time-locked changes in theta/gamma coupling as the patient was planning and imagining several movements in a row during the motor states periods. This could partly explain why the classifiers performances decreased when the time window was shortened as it becomes more probable that a window contained only motor execution without active movement planning. In a BCI context, the use of theta/gamma based features could then be useful to rapidly detect motor intention of a patient as it occurs before beta desynchronization (Combrisson et al., 2017), reducing thus the lag of the decoders. However, this hypothesis needs to be validated in further studies in which PAC dynamics could be assessed. The decrease of performances with shorter windows could also be explained by the reduction of the number of theta cycles within the windows, resulting in more uncertain PAC measures.

### 4.3 Beta/high-gamma coupling discriminates left and right hand motor states

Interestingly, another pair of PAC bands, namely beta/high-gamma, contributed to the discrimination between motor states. In particular this coupling was strongly represented in the classification of right hand vs. left hand movements. Although research on this coupling is more sparse, especially for motor decoding, several studies investigating abnormal beta/high-gamma coupling in patients with Parkinson's disease showing a deficit of its suppression, that normally occurs during movement in healthy subjects (Gong et al., 2022; Hodnik et al., 2024).

These results are in line with widely demonstrated desynchronization and fluctuations of beta oscillations during different active states of movements (Engel and Fries, 2010; Kilavik et al., 2013), which are commonly implicated in sensorimotor processing (Pfurtscheller and Lopes da Silva, 1999; Baker, 2007). Recent studies (Tan et al., 2014; Alayrangues et al., 2019; Struber et al., 2021) have strengthened the link between beta oscillations and sensorimotor processes, in particular visuo-motor error integration, by examining beta's role in motor learning contexts. Additionally, patterns of beta desynchronization have been found to be similar between motor imagery, attempted movements and actual movement, particularly in right-hand vs. left-hand movements compared to imagery or rest (Pfurtscheller et al., 2003), further underscoring the importance of beta rhythms in both actual and imagined motor tasks (Barone and Rossiter, 2021). Beta band is indeed the most commonly used characteristic of neural signals for EEG movement and motor decoding (Korik et al., 2018).

Beyond the beta band, ECoG studies, that allow for a better temporal resolution, showed that high gamma band (60 - 120 Hz) is crucial for accurate movement decoding (Moly et al., 2022; Kuo et al., 2024). However, it could have been expected that their coupling contributes to the separation between *idle* and *motor* states as suggested in studies with patients with Parkinson's disease. We hypothesize that it contributed less to this discrimination because the large widespread theta/low-gamma coupling was sufficient to dissociate them. Furthermore, the theta/low-gamma coupling being bilateral, other discriminating features may have been necessary to distinguish between motor states. The beta/high-gamma coupling, being more spatially localized distinguished motor states associated with very lateralized neural activity, suggesting that the classifier captured the suppression of the beta/high-gamma coupling within the contralateral hemisphere of each motor state.

### 4.4 PAC-based pseudo-online classification is efficient but does not outperform amplitude-based classification

The findings illustrated above could motivate the use of PAC features for online BCI. In order to investigate if PAC features provide better or different information that amplitude features, we tried to classify the three states in a pseudo-online manner. However, our results showed that PAC-based decoders—although efficient - did not completely reached the accuracy of amplitude-based decoders. Moreover, using combined features did not achieve higher performances than amplitude-based decoding. Previous regression analysis indicated however that PAC-based classifications were able to extract information not included in amplitude only. Model weights analysis also pointed in the same direction, indicating that amplitude features convey overlapped but different information than PAC features for classification. There are mainly two explanations that limited the performances of PAC-based decoding.

First, as mentioned before, the theta/low-gamma and alpha/low-gamma couplings that were the main drivers of the offline binary classifications, might be time-locked with the initiation of a movement. Using 1 s windows to compute the PAC, it is highly probable that some windows did not present any significant coupling while a movement was ongoing. This likely explains why more samples of left and right hand states were falsely classified as idle with PAC features.

Secondly, it is acknowledged that it is crucial to select an appropriate data length that corresponds to the slowest frequency being examined to obtain reliable phase-amplitude coupling measurements (Dvorak and Fenton, 2014). This is because slower oscillations produce fewer cycles within a given time frame, making it essential to have a sufficient duration of data to capture these cycles accurately (Aru et al., 2015; Tort et al., 2010). *Idle vs. motor* states classification in our study showed to be mostly driven by theta/low-gamma coupling. Theta waves are in the slower range of oscillating frequency; extracting features on a 1-second time frame, may be not enough to give meaningful information on the theta/low-gamma coupling, motivating the lower accuracy when decoding with PAC features. Conversely, beta/high-gamma coupling determines the right vs left hand classification. beta waves oscillate considerably faster than theta, making the 1-second time frame sufficient for capturing numerous complete cycles. The more reliable PAC measurement on this coupling motivates the very good distinction between left and right hand movement even in the pseudo-online setting.

These insights suggest that PAC features can correctly classify different motor states, but a balance must be found between event duration and the window length needed for accurate PAC estimation. Furthermore, a poorer distinction between RH and LH was possible using PAC features, indicating that PAC could carry less information about movement vs. movement classification than amplitude, while being able to distinguish resting state from movement.

### 4.5 Limitations and perspectives

First, it is necessary to mention that the conclusions drawn here are based on a single subject and should be validated over a larger cohort. Nevertheless, the results were consistent over 32 BCI sessions that were performed over 6 months. Only two lateralized motor tasks were considered in the main article, but we also conducted the pseudo-online analysis over the 5 states of the considered database with consistent results (see Supplementary Figure S2). Although further tasks and experiments should be investigated, this indicate that the results presented here could generalize over other tasks. In addition, binary classifications allowed reaching high decoding accuracies, and extracting significant information in coherence with the literature on PAC and oscillatory bands involved in motor processes.

Also, this study on PAC measurements for ECoG motor attempts decoding is preliminary, offering initial insights into the potential of phase-amplitude coupling as a feature for BCI. To expand on these findings, several avenues for further research can be envisioned. First, exploring alternative coupling estimation methods, such as time-resolved PAC (e.g., tPAC Samiee and Baillet, 2017), could provide finer temporal resolution without relying on predefined windows. Additionally, investigating inter-electrode coupling could help capture more complex coordination and communication between brain regions (Roehri et al., 2022), although this approach would require strong a priori hypotheses about relevant electrode pairs (e.g. sensory/motor coupling). Adapting coupling frequencies to user-specific frequency bands may also enhance decoding by tailoring the analysis to individual neural patterns.

Even though they depict information at different timescales, when using both amplitude and PAC features in pseudo online classification, tensors of PAC and amplitude were flattened and concatenated. More elaborated methods to combine and eventually select informative features before decoding should be considered to benefit from the different information provided by both features types. Model weights indeed indicated that different frequency bands and electrodes contributed to the classification between tasks, despite overlapping patterns (Figure 5C). Regression analysis also pointed out that PAC and gamma amplitude share overlapping but also distinct information. Employing more advanced non-linear classifiers could also improve decoding accuracy, potentially capturing complex patterns within PAC features.

Finally, as PAC is widely regarded as a marker of memory processes and effective communication between brain regions, observing how PAC evolves over time in a rehabilitation study could provide important perspectives, potentially revealing changes and optimization in neural communication patterns as motor functions recover.

## 5 Conclusions

In conclusion, this study provides evidence that phase-amplitude coupling (PAC) can be used as a discriminative feature for classifying motor attempts in an long-term ECoG-based BCI context. PAC features, particularly those derived from theta/low-gamma and beta/high-gamma coupling, showed the ability to distinguish between motor tasks and resting states with high accuracy in offline settings, highlighting PACs potential to capture critical motor-related neural dynamics. Despite the observed efficacy, PAC-based pseudo-online decoding did not outperform traditional amplitude-based decoding, probably due to limitations in accurately measuring coupling within short time windows. This constraint suggests a trade-off between capturing temporally brief motor events and maintaining the accuracy of PAC estimation, especially for lower frequency oscillations. As theta/low-gamma coupling is tightly time-locked to the initiation of the movement, we believe that it could be particularly suitable for BCI where motor imagery/attempts serves to trigger an event and not maintain a state as in the current study. Furthermore since theta burst occurs before beta suppression in motor tasks, theta/low-gamma coupling could be investigated to reduce delay in future BCI studies.

Future research should aim to address these challenges by refining PAC measurement techniques to enhance PAC temporal, spectral and spatial sensitivity. Additionally, further studies are needed to assess the advantages of PAC decoding, such as a possible reduction in response lag, and to explore ways in which amplitude-based decoding could benefit from incorporating PAC features. Finally, while this study highlights the potential of PAC measurements in neural oscillations to differentiate motor attempts for ECoG-BCI applications, it also provides insights into the neural components of PAC during motor processes. These findings underscore the value of rare long-term ECoG recordings to advance neurophysiological research, such as PAC in neural oscillations.

## Data Availability

The data analyzed in this study is subject to the following licenses/restrictions: The data analyzed during the current study are not publicly available for legal/ethical reasons. Part of the dataset may be provided upon reasonable request. Requests to access these datasets should be directed to LS, lucas.struber@cea.fr.
