# Supplementary material for: Classifying mental motor tasks from chronic ECoG-BCI recordings using phase-amplitude coupling features
Source: Front Hum Neurosci. 2025 Mar 12;19:1521491. doi: 10.3389/fnhum.2025.1521491 (PMC11936922; doi:10.3389/fnhum.2025.1521491)
Supplement: Supplementary file 1 [file Data_Sheet_1.pdf]

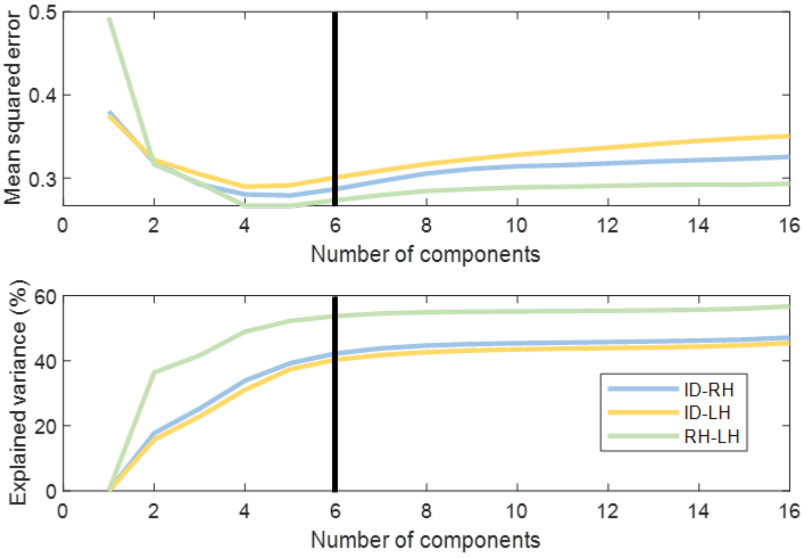

Figure S 1. Evolution of mean squared error and explained variance depending on the number of PLS components used, for the three considered classification. This analysis was performed on sessions recorded before the sessions presented in the main text.

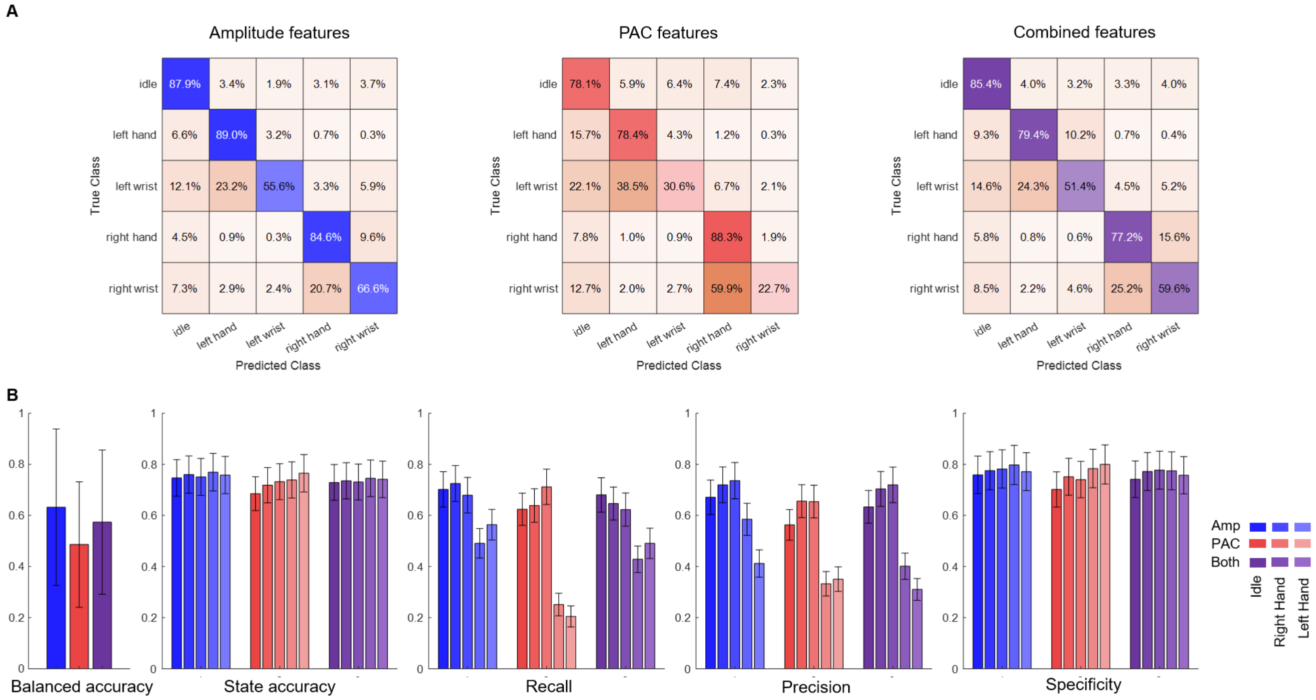

Figure S 2. Pseudo online classification results for 5 states classification.(A) Confusion matrices (row normalized) using the different input features (B) Average balanced accuracy, accuracy (one versus rest), recall, precision and specificity. Except for balanced accuracy, metrics were calculated per state. Performances of the pseudo-online classification are shown for amplitude (blue), PAC (red) or both (purple) features on the three states classification. States are shown in different shades.
